# The effectiveness and cost-effectiveness of 3- vs. 6-monthly dispensing of antiretroviral treatment (ART) for stable HIV patients in community ART-refill groups in Zimbabwe: study protocol for a pragmatic, cluster-randomized trial

**DOI:** 10.1186/s13063-018-2469-y

**Published:** 2018-01-29

**Authors:** Geoffrey Fatti, Nicoletta Ngorima-Mabhena, Frank Chirowa, Benson Chirwa, Kudakwashe Takarinda, Taurayi A. Tafuma, Nyikadzino Mahachi, Rudo Chikodzore, Simon Nyadundu, Charles A. Ajayi, Tsitsi Mutasa-Apollo, Owen Mugurungi, Eula Mothibi, Risa M. Hoffman, Ashraf Grimwood

**Affiliations:** 1 Kheth’Impilo AIDS Free Living, 11th floor, Metlife Centre, 7 Walter Sisulu Ave, Cape Town, 8000 South Africa; 2 Kheth’Impilo AIDS Free Living, 7 Albany Road, Alexandra Park, Harare, Zimbabwe; 3 International Union against Tuberculosis and Lung Disease, Paris, France; 4 AIDS and TB Department, Zimbabwe Ministry of Health and Child Care, 2nd Floor, Mkwati Building, Corner Livingstone Avenue and 5th Street, Harare, Zimbabwe; 5 FHI360-Zimbabwe, 65 Whitwell Rd, Borrowdale West, Harare, Zimbabwe; 6 Zimbabwe Ministry of Health and Child Care, Matabeleland South Provincial Medical Directorate, First Floor New Government Complex, Third Avenue, Gwanda, Zimbabwe; 7 Zimbabwe Ministry of Health and Child Care, Midlands Provincial Medical Directorate, Gweru, Zimbabwe; 8 Health, Population and Nutrition Office, United States Agency for International Development- Zimbabwe, 1 Pascoe Avenue, Belgravia, Harare, Zimbabwe; 9 College of Health Sciences, University of Zimbabwe, Harare, Zimbabwe; 10 Department of Medicine, Division of Infectious Diseases, David Geffen School of Medicine, University of California, 10833 Le Conte Ave 37-121 CHS, Los Angeles, CA 90095 USA

**Keywords:** HIV, Antiretroviral treatment, Multimonth dispensing, Extended dispensing interval, Community ART-refill groups, Zimbabwe, Effectiveness, Cost-effectiveness, Cluster-randomized trial, Sub-Saharan Africa

## Abstract

**Background:**

Sub-Saharan Africa is the world region with the greatest number of people eligible to receive antiretroviral treatment (ART). Less frequent dispensing of ART and community-based ART-delivery models are potential strategies to reduce the load on overburdened healthcare facilities and reduce the barriers for patients to access treatment. However, no large-scale trials have been conducted investigating patient outcomes or evaluating the cost-effectiveness of extended ART-dispensing intervals within community ART-delivery models. This trial will assess the clinical effectiveness, cost-effectiveness and acceptability of providing ART refills on a 3 vs. a 6-monthly basis within community ART-refill groups (CARGs) for stable patients in Zimbabwe.

**Methods:**

In this pragmatic, three-arm, parallel, unblinded, cluster-randomized non-inferiority trial, 30 clusters (healthcare facilities and associated CARGs) are allocated using stratified randomization in a 1:1:1 ratio to either (1) ART refills supplied 3-monthly from the health facility (control arm), (2) ART refills supplied 3-monthly within CARGs, or (3) ART refills supplied 6-monthly within CARGs. A CARG consists of 6–12 stable patients who meet in the community to receive ART refills and who provide support to one another. Stable adult ART patients with a baseline viral load < 1000 copies/ml will be invited to participate (1920 participants per arm). The primary outcome is the proportion of participants alive and retained in care 12 months after enrollment. Secondary outcomes (measured at 12 and 24 months) are the proportions achieving virological suppression, average provider cost per participant, provider cost per participant retained, cost per participant retained with virological suppression, and average patient-level costs to access treatment. Qualitative research will assess the acceptability of extended ART-dispensing intervals within CARGs to both providers and patients, and indicators of potential facility-level decongestion due to the interventions will be assessed.

**Discussion:**

Cost-effective health system models that sustain high levels of patient retention are urgently needed to accommodate the large numbers of stable ART patients in sub-Saharan Africa. This will be the first trial to evaluate extended ART-dispensing intervals within a community-based ART distribution model, and results are intended to inform national and regional policy regarding their potential benefits to both the healthcare system and patients.

**Trial registration:**

ClinicalTrials.gov, ID: NCT03238846. Registered on 27 July 2017.

**Electronic supplementary material:**

The online version of this article (10.1186/s13063-018-2469-y) contains supplementary material, which is available to authorized users.

## Background

Sub-Saharan Africa carries the highest burden of HIV, with approximately 70% of all people living with HIV globally found in this region [1]. In 2016, the World Health Organization (WHO) broadened its guidelines to recommend that all identified people living with HIV should initiate antiretroviral treatment (ART) as soon as possible after diagnosis, irrespective of clinical or immunological status [2]. This policy is expected to reduce AIDS-related mortality, morbidity and new HIV infections. It does, however, substantially impact already overburdened health systems in sub-Saharan Africa which need to accommodate substantially increased numbers of ART patients at a time when resources for HIV are constrained globally and there is a severe shortage of professional health workers [3, 4]. An important component of the current research agenda is the development of systems that improve the efficiency of healthcare delivery by reducing unnecessary burdens on the healthcare system and better serve the needs of the large numbers of people living with HIV [5].

To realize the benefits of lifelong ART, high levels of adherence and retention are required. Barriers to adherence and retention include long waiting times at ART clinics, cost of travel to clinics, and life events that cause treatment interruptions [6–9]. Needing to frequently collect ART refills (every 1–2 months) may lead to suboptimal adherence and disengagement from care due to the time and cost to patients of frequent clinic visits, particularly for those who have to travel long distances [10, 11]. Frequent clinic visits also place a high demand on the healthcare system due to the costs of providing personnel and infrastructure. Modeling has shown that reducing the frequency of clinic visits for stable patients is expected to be cost-effective in sub-Saharan Africa [12]. Clinics that are decongested of stable ART patients may also find place to increase the rate of new ART initiations and thus scale up ART coverage [13].

Observational research in Zambia has shown that 2- and 3-monthly visit intervals were associated with decreased loss to follow-up (LTFU) and decreased visit lateness compared to patients who attended monthly [14]. A recent systematic review found that reduced frequency of clinic visits and medication pick-up for ART patients may lead to improvements in program retention and patient outcomes [15]. As a result, the WHO has recommended that stable ART patients require less frequent medication pick-ups and clinic visits (3- to 6-monthly) [2, 5]. However, the available data is sparse and the quality of evidence is “very low to low due to bias” [15]. A single, pilot cluster-randomized trial that included a comparison of clinic visit frequencies (maximum 3-monthly) for ART patients has been conducted in low-income settings, but included only 96 participants in the intervention group [16]. No larger-scale randomized trials have been conducted, and little cost data is available.

In Zimbabwe, almost 1.5 million people require ART and the country initiated more than 9000 people on ART each month in 2015 [17]. The Zimbabwe Ministry of Health and Child Care (MOHCC) has suggested that innovative strategies are required to achieve ART program goals without overwhelming the health system and to maintain a quality service [7]. Community ART-refill groups (CARGs) are a novel strategy that has been introduced with the intent to reduce barriers to patients accessing regular treatment and to limit health facility congestion [18]. CARGs are groups consisting of 6–12 stable ART patients who meet at a community venue to receive ART refills, and who provide mutual support to each other. Retrospective observational studies of community groups receiving monthly ART refills have shown encouraging results [19, 20]; however, no randomized trials have been conducted investigating prolonged ART-dispensing intervals within CARGs. Implementation research is needed to inform on the expected benefits of differentiated care models for stable ART patients in terms of their clinical and cost-effectiveness [5].

The aim of this study is to compare the clinical effectiveness and cost-effectiveness of three models of ART provision for stable ART patients in Zimbabwe, with medication pick-ups extended up to 6 months. The objectives are to measure patient retention, virological suppression, acceptability and provider and patient costs amongst stable patients who receive ART at 3- and 6-monthly intervals within CARGs. These groups will be compared to standard of care, facility-based, 3-monthly ART delivery.

## Methods

### Study design

This study is a pragmatic, three-arm, parallel, unblinded, cluster-randomized non-inferiority trial using stratified randomization with 24 months of participant follow-up. This trial is operational research based on the Zimbabwe Operational Service Delivery Manual for HIV prevention, care and treatment which outlines guidelines for differentiated service delivery for stable ART patients [18]. The study protocol was developed using the Standard Protocol Items: Recommendations for Interventional Trials (SPIRIT) Checklist (see Additional file 1) and adheres to the SPIRIT recommendations. Each arm will consist of 10 clusters (health facilities) as follows:

- Control arm: participants will receive ART at 3-month intervals provided at the facility (arm 3MF)
- Intervention arm 1: participants will receive ART at 3-month intervals provided in CARGs (arm 3MC)
- Intervention arm 2: participants will receive ART at 6-month intervals provided in CARGs (arm 6MC)

### Outcomes and hypotheses

The rationale for the control arm selection is that provision of ART refills 3-monthly at the facility is the most established standard of care option for stable ART patients in Zimbabwe. All outcomes will be compared between all three arms. The primary outcome is participant retention in care, defined as the proportion of participants remaining in care 12 months after enrollment by intention-to-treat. Retention in HIV care is one of the most important determinants of the overall impact of ART [21]. Retention in care will also be compared after 24 months.

The principal hypotheses are that participant retention in both the intervention arms will be non-inferior to retention in the control arm with a non-inferiority margin of − 3.25% (risk difference). An additional hypothesis is that retention in intervention arm 6MC will be non-inferior to retention in intervention arm 3MC. The rationale for the non-inferiority design is that we anticipate retention to be at least similar in the intervention arms vs. control, and anticipate that provider (and participant-level) costs will be lower in the intervention arms vs. the control due to reduced participant contact with healthcare facilities in the intervention arms. If clinical outcomes in the intervention arms are at least non-inferior compared to the control arm, the interventions are anticipated to show cost savings compared to the control.

The secondary outcomes are:

I. The proportion of participants achieving virological suppression 12 and 24 months after study enrollment
II. The proportion of participants retained in the study model of care after 12 and 24 months
III. Average annual cost per participant from a provider perspective
IV. Average provider cost per participant retained, and average provider cost per participant retained with virological suppression 12 and 24 months after enrollment
V. Average annual participant-level expenditure to access treatment
VI. Qualitative research to assess the acceptability of multimonth dispensing (MMD) of ART within CARGs from both a participant and healthcare provider perspective
VII. Site-level indicators of potential facility-level decongestion during the study. These are:
  - Median difference in facility patient waiting times after 12 and 24 months compared to baseline
  - Trend in the monthly number of patients newly initiated on ART
  - Trend in the monthly number of patients who receive provider-initiated HIV counseling and testing at the clinic

### Definitions

The primary outcome of retention in care will be defined as 1-participant attrition, where attrition is defined as either death (all-cause) or loss to follow-up (LTFU). LTFU will be defined as no facility or ART collection for > 90 days after the last missed scheduled ART collection [22, 23]. (Participants not arriving for the 12- or 24-month visit will be considered retained at these time points if collecting ART in person within 90 days of the scheduled appointment date). Participants with documented transfers to another clinic will be considered retained at the next immediate time point (12 or 24 months), and will then be censored. For the secondary outcome of retention in the study model of care, participants will be considered non-retained if transitioning off the study arm for any reason, including death, LTFU, change in ART-dispensing frequency, incident comorbidity requiring more frequent clinic visits, personal preference or withdrawal of consent to participate. Reasons for a change in ART-dispensing frequency include incident opportunistic infections, drug toxicity requiring closer monitoring, provider judgment based on change in clinical status or inadequate adherence, viral load > 1000 copies/ml, or pregnancy. Viral suppression will be defined as a viral load < 1000 copies/ml.

### Description of interventions

The study implementing partners employing the CARG model will align to the routine Zimbabwe CARG model [24], with adaptations to allow for extended ART-dispensing intervals. CARGs consist of 6–12 people, and CARG participants live in a similar geographic location and attend the same health facility. Participants at facilities in the 3MC and 6MC arms will be enrolled from newly formed CARGs (under 3 months) in which members have not had their first CARG-refill meeting. These CARG members will receive a viral-load test to ascertain stability and eligibility for the study. A CARG leader will be nominated, and the CARG will meet on at least a 3-monthly basis at a venue of their choice to access treatment. They may meet more regularly pending the group need for adherence support or to address psychosocial barriers to adherence. Stable study participants will have a clinical consultation and viral-load test at the facility at least annually after enrollment. Thus, after 12 months and 24 months, the entire CARG will be required to report to the facility on the same day. Each CARG member will collect their own ART supply on this day. For the 3MC arm, a single, alternating CARG representative will report to the facility after 3, 6, 9, then 15, 18 and 21 months to collect a 3-month supply of ART for all members of the CARG. This CARG representative will distribute the medicines to the other CARG members at the CARG meeting on the same or the following day. For the 6MC arm, a single CARG representative will report to the facility at 6 months and again after 18 months to collect a 6-month ART supply for all members of the CARG. Each CARG will be supplied with a back pack or small wheeled suitcase to carry medicines. Participants in the control arm (3MF) will receive their own supply of ART collected 3-monthly at the facility, and will receive annual clinical consultations.

### Setting and site selection

The study facilities are public health facilities located in Chitungwiza municipality, Masvingo and Matebeleland South provinces and Mberengwa district. These areas are representative of high-HIV-burden districts prioritized for scale-up of HIV services with increased support from the US President’s Emergency Plan for AIDS Relief (PEPFAR), and health facilities in these areas have prioritized the implementation of CARGs. Chitungwiza municipality is an urban local authority situated about 25 km south of the capital city, Harare. Masvingo and Matebeleland South are predominantly rural provinces located in the south-eastern and south-western regions of Zimbabwe, respectively, while Mberengwa is a rural district in Midlands province.

We selected 30 facilities from the study districts according to the following site selection criteria: (1) supply-chain procedures for implementation of MMD of ART at the site were deemed to be feasible by the facility managers and MOHCC, (2) implementation of CARGs were deemed to be feasible or had recently been implemented, (3) routine site-data collection systems were adequate and (4) at least 430 adults were receiving ART (to fulfill site sample-size requirements).

### Cluster allocation

**Fig. 1:**
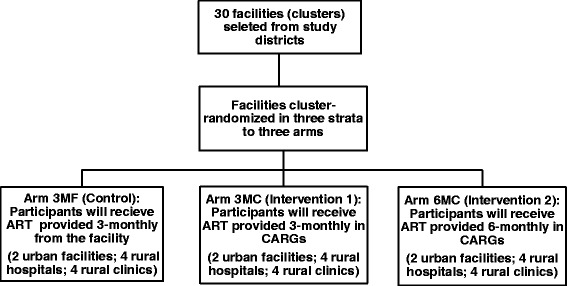
Cluster allocation schema of multimonth dispensing of antiretroviral treatment cluster-randomized trial in Zimbabwe. *CARGs* community antiretroviral treatment-refill groups

Figure 1 illustrates the cluster allocation schema. To produce balance in urban and rural facilities as well as hospitals and clinics between the arms, a restricted randomization using three strata has been conducted [25], i.e., urban facilities (six facilities); rural hospitals (12 facilities) and rural clinics (12 facilities). One large rural clinic which has a large patient load similar to the rural hospitals, and which is currently applying to become a hospital, will be considered a hospital for the study. Following the stratified randomization, each arm consists of two urban facilities, four rural hospitals and four rural clinics. Cluster randomization has been conducted by an independent statistician using random-number generation with Microsoft Excel®.

### Participant eligibility criteria

Participant eligibility will be aligned to those for stable ART patients in routine settings in Zimbabwe [24], and are shown in Table 1.

**Table 1:** Inclusion and exclusion criteria for study participants

Inclusion criteria	Exclusion criteria
18 years of age or older	ART toxicity or tolerability issues in the prior 6 months
Confirmed HIV-1 infection based on the Zimbabwe national HIV-testing algorithm	Treatment for an opportunistic infection in the prior month
Received standard first-line ART for 6 months or longer	Receiving alternative first-line or second-line ART regimen
Viral load < 1000 copies/ml at enrollment	Active or suspected opportunistic infection
Weight ≥ 35 kg	Active or suspected tuberculosis
Willing to join a CARG	Comorbidities requiring more frequent visits to the facility than that required by the model of care
Provide informed consent for inclusion in the study	Confirmed pregnancy
	Less than 18 months postpartum

*ART* antiretroviral treatment, *CARG* community antiretroviral treatment-refill group, *HIV* human immunodeficiency virus

### Sample size estimation

Participant sample size estimates were calculated for the primary outcome of retention in care 12 months after enrollment. Sample size estimates were determined for a non-inferiority test for the difference in two proportions in a cluster-randomized design, using PASS® v.14 software. Participant enrollment numbers are assumed to be equal at each cluster. The probability of participant retention 12 months after study enrollment in the control group is assumed to be 95%, derived from the relative difference in reported retention between 12 and 24 months of ART in Zimbabwe [17]. The probability of retention in the intervention arms after 12 months is also assumed to be 95%. An intracluster correlation coefficient of 0.01 for retention amongst stable ART patients associated with the same healthcare facility is assumed [26]. The non-inferiority margin is prespecified as − 3.25% (risk difference). Assuming *α* = 0.05, power of 85%, and using the one-sided Z-test (unpooled) statistic, 192 participants will be required to be enrolled per facility, with 1920 participants per arm and a total sample size of 5760 enrolled participants. As retention in care is the primary outcome, no adjustment for LTFU is made. Assuming that 75% of those on ART at a healthcare facility will be stable [17] and that 60% of these will consent to be enrolled, to reach the required sample size per facility, selected facilities require at least 430 adults currently receiving ART.

### Recruitment and enrollment procedures

**Fig. 2:**
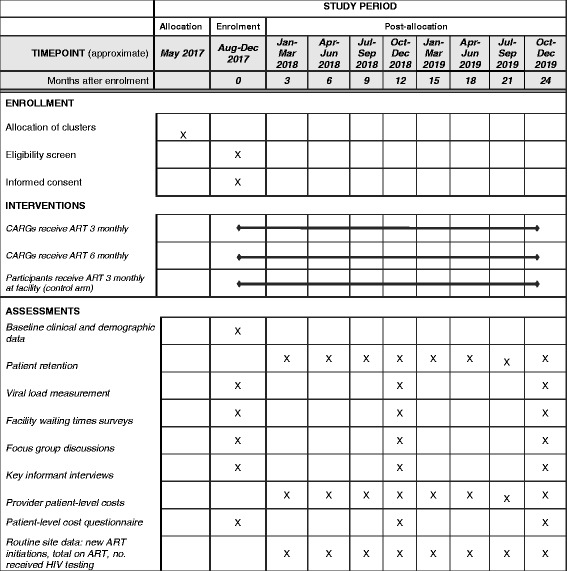
Enrollment, interventions and assessment schedule according to Standard Protocol Items: Recommendations for Interventional Trials (SPIRIT)

Figure 2 illustrates the overview of time schedules for enrollment, interventions and assessments according to SPIRIT. During the enrollment period, study nurses will screen all patients arriving for ART-refill visits at the study facilities and patients in recently formed CARGs according to the eligibility criteria. Potentially eligible patients will be invited by the study nurses to participate in the study, and to receive viral-load testing. It is not mandatory for all patients in a CARG to participate in the study. Eligible patients at each site who provide informed consent and who have a viral load < 1000 copies/ml will be enrolled until the facility enrollment cap has been reached. All study participants at a particular facility will receive the same model of care based on the arm to which that facility has been allocated. Patients already in CARGs at facilities in the control arm at the commencement of the study will not be considered for recruitment, and they will continue to receive care in CARGs as per the national guidelines similar to those in the 3MC arm.

### Participant follow-up

All participants will be followed up for 24 months. As retention in care is the primary outcome, there will be no involvement regarding defaulter tracking beyond which exists routinely at each facility. Viral-load testing will occur at annual intervals, according to national guidelines [18]. Participants who become pregnant, develop tuberculosis or other opportunistic infections, have unsuppressed viral loads (≥1000 copies/ml), or who become clinically unwell will require clinic follow-up visits more frequently than 3-monthly as per national guidelines, thus they will transition off the study arm. These participants will remain under observational follow-up for the remainder of the study period for ascertainment of the primary outcome. CARG participants will return to be followed up at the facility. Frequency of ART-dispensing and clinical management will then be determined based on clinician assessment and national guidelines.

As Zimbabwe is moving to have ART refills provided in CARGs as an additional option for stable ART patients at all facilities [18], participants in the 3MF arm will be consigned to receive ART 3-monthly supplied at the facility for 12 months after enrollment; thereafter, participants will be offered the option of forming CARGs that receive ART 3-monthly. Participants who decline to join CARGs will continue to be followed up as arm 3MF for a further 12 months. This design was chosen taking into account program implementation plans for PEPFAR partners as they prioritize the roll-out of CARGs. It will allow arm 3MF to have at least 12 months of follow-up time to analyze retention and virologic suppression. After 24 months, all participants will receive nationally applied standard of care as at the time of study completion.

### Data collection

The study will use a mixed-methods approach with collection of individual-level quantitative, qualitative, provider and participant-cost data, as well as site-level outcome data.

### Individual-level quantitative data collection

The study electronic database for data collection and management has been developed using REDCap (Research Electronic Data Capture) electronic data capture tools [27]. The Zimbabwe MOHCC data collection tools at facilities and CARGs will be used for source data collection including the patient file, the routine electronic patient management system for HIV patients, and routine CARG data collection forms. Trained study-specific nurses will extract source data and capture these with electronic tablet devices following participant CARG or clinic visits. Study nurses will also follow up results of viral-load tests. The data manager will perform regular range checks and missing value checks in the database. On a 3-monthly basis, the study data manager will conduct quality assurance by conducting 100% manual verification checks on a 5% random sample of study participant files of each data capturer’s entries with source data.

### Qualitative data collection

Qualitative data will be collected by trained nurse facilitators at baseline, after 12 months and at the end of the study to understand the acceptability of MMD of ART within CARGs to patients and service providers, as well as to evaluate potential improvements in the quality of service delivery at facilities due to decongestion of facilities. Data collection methods will include focus group discussions (FGD) with study participants and key informant interviews (KII) with facility managers and healthcare workers. One FGD per facility consisting of up to 10 randomly selected CARG participants will be conducted in the vernacular, with 20 FGDs held at each data collection point. Twenty KIIs will be performed at each data collection point. All qualitative data will be recorded, transcribed, translated to English and coded.

### Cost data collection

A micro-costing approach will be used, and a macro-costing approach of fixed costs will be utilized. To ascertain provider costs, both fixed and variable costs will be estimated. Fixed costs will include buildings, equipment, vehicles, CARG supervision and start-up costs, and administration costs. Variable costs for each patient will include outpatient and inpatient care, antiretroviral and non-antiretroviral drug costs, consumables, laboratory tests and components, human resource time per patient, salary cost per patient per service provided and overhead costs including telephone calls and home visits. A resource-use form will be used to collect patient-level costs from a review of clinic records including clinic notes, pharmacy registers, laboratory records and hospital inpatient records. A separate data collection tool will be used to retrospectively collect fixed costs’ information.

Patient-level cost questionnaires will be administered at baseline, 12 months and at 24 months using a pretested data collection tool on a subset of participants (*n* = 1095). Every fifth participant enrolled consecutively per site will be selected for administration of the patient-level cost questionnaires. These costs will include those borne by either the patient or the patient’s relatives to access care. Patient time and accounting for potential loss of income for attending CARG meetings will be incorporated in patient-level costs.

### Site-level data collection

A pre, 12-month and 24-month time-flow survey of patient waiting time over 5 days each will be conducted at each facility using a time-flow recording sheet for each clinic patient. The monthly number of people newly initiated on ART and the monthly number of people who receive provider-initiated HIV testing and counseling per facility will be sourced from routine facility records.

### Data analysis plan

#### Quantitative patient-level data analysis

Descriptive measures of the study population at baseline in each study arm will be conducted using medians and interquartile ranges for continuous variables and proportions for categorical variables, to assess potential imbalances between the arms.

Individual-level outcome analyses will be conducted by intention-to-treat. For the primary outcome of patient retention, risk differences between arms will be estimated using binomial population-averaged generalized estimating equations using an identity link and an exchangeable correlation structure, specifying for clustering by facility. A small cluster size variance correction will be used, and randomization strata will be included in the model as a fixed-effect parameter. If the lower bound of the 95% confidence interval for the risk difference in patient retention of the intervention vs. control arm is greater than the non-inferiority limit of − 3.25%, the intervention will be considered non-inferior to the control. Where there are imbalances between study arms in baseline variables, these will be adjusted for in multivariable analyses.

For the outcome of viral suppression, log-binomial regression with generalized estimating equations will be used to estimate risk ratios between the study arms, specifying for clustering by facility. An exchangeable correlation structure will be used and a small cluster size variance correction applied. Viral-load suppression analyses will be conducted according to: (1) an intention-to-treat principle including all enrolled participants in the arm to which they were originally allocated and irrespective of whether they had available follow-up viral-load results or completed the study and (2) a per-protocol analysis including only patients with available viral-load results. If there are imbalances between study arms in baseline variables, these will be adjusted for in multivariable analyses. Subgroup analyses will include comparing outcomes by strata of urban or rural facility and by gender. A pooled analysis of both intervention arms vs. control will also be performed.

#### Qualitative data analysis

Content qualitative analysis will be employed. Summative content analysis involving counting and comparisons of keywords or content followed by the interpretation of the underlying context will be conducted. Atlas Ti ® software will be used to assist in coding and locating particular words or phrases from the transcribed interviews and discussions. A deductive coding frame to extract code frequencies will be developed. The data will be analyzed for emerging key themes and the findings will be interpreted based on the research questions. Quotes will also be extracted for each of the themes that emerge from the data.

### Cost analysis

The study will utilize the concept and methodology used in Zambia regarding retention in care and outpatient costs for children on ART [28]. We will estimate the total annual costs of fixed costs and use an appropriate measure to allocate them on a pro-rata basis. This will be achieved by recording the number of patients that visit the study facilities (apart from HIV patients) during the study period, that will enable fair allocation of fixed costs per patient. This will be done for all shared resources within the facility. Buildings and equipment costs will be estimated using a replacement approach and annualized. The annual cost will be divided by the total number of active patients in that year for each facility. The administrative cost contribution per patient will be calculated by dividing the annual salaries for administrative staff by the number of active patients for each year for each facility.

Total costs for each arm will be disaggregated into two, namely (1) total costs including start-up costs to capture costs that would have been introduced by the study and (2) costs excluding start-up costs to capture routine cost of care.

A cost outcomes analysis will be conducted from a provider perspective for each arm at 12 and 24 months. The total cost for each arm will be calculated as the sum of the fixed and variable unit costs, and the average cost per patient per year for each arm will be calculated. The average cost per patient retained in care and average cost per patient virally suppressed will be calculated at 12 months and 24 months.

The patient-level cost analysis will involve comparing the baseline average patient costs with patient costs reported at 12 and 24 months for each arm, and comparing costs between arms. The analysis will consider number of visits made to the facility and lost potential production time.

### Site-level data analysis

Summary measures of patient waiting time for each facility at each assessment point will be calculated. The difference in waiting time at 12 and 24 months vs. the baseline survey will be calculated within each arm, and differences in waiting time will be compared between arms. In addition, between-arm comparisons of 12- and 24-month waiting times will be conducted with a two-level, mixed-effects linear model including the median baseline facility waiting time as a fixed covariate and specifying facility as a random effect. A secondary analysis will involve inclusion of other site-specific potential confounding variables as fixed effects in the model.

Plots will be drawn of the monthly numbers of patients newly initiated on ART and monthly numbers who receive provider-initiated HIV counseling and testing per site for each arm to assess trends during the study period, and multilevel, mixed-effects linear regression will be used to compare arms. A secondary analysis will include adjustment for potential site-specific confounding.

### Study monitoring

Site monitors will visit study sites for inspections and to review participant study records including consent forms and data collection tools. An independent Data and Safety Monitoring Board (DSMB) consisting of three members has been appointed to monitor the trial, one of whom is a biostatistician. The DSMB members do not have competing interests. The DSMB will advise on any adjustments required to achieve balance in the trial (expand sites or replace sites) during accrual. Interim analyses will be conducted when 50% of the participants have completed 12 months on the study. From the results of interim analyses, the DSMB will consider terminating the trial if retention at 12 months in any of the arms is highly significantly different to the other arms (*p* value < 0.01). The DSMB may also choose to stop the study early for highly significant, and by the judgment of the DSMB, clinically important differences in outcomes.

### Ethical and regulatory considerations

This study has received ethical approval from the Medical Research Council of Zimbabwe, and approvals from the Research Council of Zimbabwe and the Ministry of Health and Child Care of Zimbabwe.

### Confidentiality

All study procedures will be conducted in private and every effort will be made to protect participants’ privacy and confidentiality. At study sites, participant research records will be stored in locked areas with access limited to study staff. Participants will be identified in the study database with an anonymous identifier only. All participant electronic data will be password protected.

### Dissemination of results

The study results will be communicated via reports to the Zimbabwe MOHCC, implementation partners and the study facility managers. In addition, the results will be submitted for publication in peer-reviewed scientific journals and presented at scientific conferences.

## Discussion

No large-scale trials have been conducted investigating patient-level outcomes and health system costs of extended ART-dispensing intervals for patients within community-based groups in sub-Saharan Africa. The rationale for this study is to provide evidence regarding the effectiveness and cost-effectiveness of extended dispensing intervals for ART patients within CARGs, and whether these are suitable strategies that can be implemented on a larger scale to help overburdened health facilities with the large numbers of patients and reduce the burden and cost to patients of frequent clinic visits. Study results are expected to inform health policy both in Zimbabwe and in the entire sub-Saharan African region.

The trial is implemented through a collaboration between Kheth’Impilo AIDS Free Living, EQUIP Innovation for Health, the Organization for Public Health Interventions and Development, FHI 360, the Zimbabwe AIDS Prevention Project Trust and the Zimbabwe MOHCC. Implementation challenges include large distances between facilities, limited access to routine viral-load testing within the country and limited Internet connectivity in rural areas.

The strengths of the study include that a variety of outcome data will be analyzed including clinical effectiveness data, provider- and patient-cost data, qualitative data and site-level impact data. Routine ART facilities from both urban and rural areas are included in a pragmatic design, which is expected to allow study results to be generalizable to other sub-Saharan African settings. The study limitations include that participants in the 3MF arm may not complete 24 months’ follow-up in this arm as, according to MOHCC operational recommendations, they will be provided the option of forming CARGs after 12 months of study participation. The sample size in the 3MF arm may thus be reduced at the 24 months’ outcomes assessment, thus comparisons may have reduced power. However, this will not impact the primary outcome ascertainment of patient retention 12 months after enrollment. Participant viral-loads results may not be complete due to limitations in the availability of viral-load testing in the country. Viral suppression is thus a secondary outcome. It will also not be possible for participants or providers to be blinded as to the study group allocation.

Vast scale-up of ART-delivery services are required for sub-Saharan Africa to reach the UNAIDS goals of 90% of people with diagnosed HIV infection receiving sustained ART and 90% of all people receiving ART having viral suppression [29]. Efficient use of limited resources incorporating community-based service delivery models may be important components of this scale up. This will be the first trial to evaluate extended ART-dispensing intervals within a community-based ART distribution model, and seeks to provide high-quality evidence within programmatic settings of a high-HIV-prevalence region to inform policy regarding efficient health systems models to support the large numbers of people requiring ART.

### Trial status

Protocol version 6.7, 16 July 2017. Recruitment commenced on 28 July 2017 and is ongoing; expected completion of recruitment is approximately the end of December 2017.

## Additional file

Additional file 1: Standard Protocol Items: Recommendations for Interventional Trials (SPIRIT) 2013 Checklist: recommended items to address in a clinical trial protocol and related documents. (DOC 121 kb)

## Abbreviations

3MC: Participants will receive antiretroviral treatment provided 3-monthly in community antiretroviral treatment-refill groups
3MF: Participants will receive antiretroviral treatment provided 3-monthly from the facility
6MC: Participants will receive antiretroviral treatment provided 6-monthly in community antiretroviral treatment-refill groups
ART: Antiretroviral treatment
CARG: Community antiretroviral treatment-refill group
DSMB: Data and Safety Monitoring Board
FGD: Focus group discussion
KII: Key informant interview
LTFU: Loss to follow-up
MMD: Multimonth dispensing
MOHCC: Ministry of Health and Child Care of Zimbabwe
PEPFAR: United States President’s Emergency Plan for AIDS Relief
REDCap: Research Electronic Data Capture
SPIRIT: Standard Protocol Items: Recommendations for Interventional Trials
WHO: World Health Organization
